# Untargeted Metabolomic Profiling of Aqueous and Lyophilized Pooled Human Feces from Two Diet Cohorts Using Two-Dimensional Gas Chromatography Coupled with Time-of-Flight Mass Spectrometry

**DOI:** 10.3390/metabo13070828

**Published:** 2023-07-07

**Authors:** Seo Lin Nam, Kieran Tarazona Carrillo, A. Paulina de la Mata, James J. Harynuk

**Affiliations:** Department of Chemistry, University of Alberta, Edmonton, AB T6G 2G2, Canada; seolin@ualberta.ca (S.L.N.); ktarazon@ualberta.ca (K.T.C.); delamata@ualberta.ca (A.P.d.l.M.)

**Keywords:** feces, metabolomics, data analysis, GC×GC-TOFMS, lyophilization, aqueous fecal

## Abstract

The metabolic profiles of human feces are influenced by various genetic and environmental factors, which makes feces an attractive biosample for numerous applications, including the early detection of gut diseases. However, feces is complex, heterogeneous, and dynamic with a significant live bacterial biomass. With such challenges, stool metabolomics has been understudied compared to other biospecimens, and there is a current lack of consensus on methods to collect, prepare, and analyze feces. One of the critical steps required to accelerate the field is having a metabolomics stool reference material available. Fecal samples are generally presented in two major forms: fecal water and lyophilized feces. In this study, two-dimensional gas chromatography coupled with time-of-flight mass spectrometry (GC×GC-TOFMS) was used as an analytical platform to characterize pooled human feces, provided by the National Institute of Standards and Technology (NIST) as Research-Grade Test Materials. The collected fecal samples were derived from eight healthy individuals with two different diets: vegans and omnivores, matched by age, sex, and body mass index (BMI), and stored as fecal water and lyophilized feces. Various data analysis strategies were presented to determine the differences in the fecal metabolomic profiles. The results indicate that the sample storage condition has a major influence on the metabolic profiles of feces such that the impact from storage surpasses the metabolic differences from the diet types. The findings of the current study would contribute towards the development of a stool reference material.

## 1. Introduction

Metabolomics is the comprehensive study of small molecules (<1500 Da) in biological samples commonly known as metabolites, and the complete set of all the metabolites is referred as the metabolome [1,2]. Studies have shown that a combination of genetic and environmental factors can influence the metabolome [3,4,5]. Organisms’ responses to conditions such as disease or environment alter their metabolic profiles, and the resulting changes can be detected in biological samples, which reveal information indicative of the host’s state of health [6,7]. Vast applications in metabolomics were achieved using numerous biological matrices, which include blood, urine, feces, saliva, sweat, breath, and tissue [8,9,10,11,12,13,14,15,16,17]. Of these biological sample types, feces is a vital source for unraveling intricate biological processes of host–microbiome interactions in a non-invasive way [18,19].

To truly access and exploit the information feces presents, analytical challenges in studying feces need to be appropriately addressed [19,20,21]. Feces, a solid waste product containing undigested food residues, has a highly complex, dynamic, and heterogeneous nature [19]. Therefore, pre-analytical aspects, including sample storage and preparation, are crucial to ensure sample integrity is maintained and unintended post-collection changes within a sample are avoided. These changes are due to ongoing processes such as oxidation, degradation, and bacterial or enzymatic activities [22,23,24]. Despite the awareness of the importance of sample handling for feces, there is currently no consensus on how fecal samples should be handled prior to analysis for unbiased stool metabolomics [25,26].

Using fresh stool samples immediately after collection (within an hour) is usually unrealistic, especially for large-scale metabolomics studies. To store samples until analysis, two sample conditions are commonly used: lyophilized feces [26,27] and fecal water (aqueous phase) [28,29,30]. Different sample conditions may affect the composition and metabolic profile of the sample. Consequently, they may influence the discovery of biomarkers and the characterization of metabolic phenotypes. However, to the best of the authors’ knowledge, a systematic study has not been conducted to compare the two sample conditions generally used in stool metabolomics.

Most fecal metabolomics studies have been carried out using mass spectrometry (MS)-based techniques and nuclear magnetic resonance (NMR) spectroscopy [20,21,31,32,33]. For MS-based platforms, they are generally coupled with a high-performance separation instrument such as gas chromatography (GC) or liquid chromatography (LC) [21,27,32,34]. Amongst many analytical platforms, GC×GC-TOFMS is a well-suited tool for untargeted studies of complex metabolomics samples [1,26,35,36,37]. GC×GC employs two independent separation mechanisms with different chemistries and offers many analytical advantages compared to a conventional one-dimensional GC system. It provides enhanced sensitivity and dynamic range with an order-of-magnitude increase in separation capacity and can routinely detect thousands of compounds in a single sample.

Herein, pooled human stool samples from two cohorts (omnivore and vegan), stored in two conditions (lyophilized and aqueous), were evaluated to compare the metabolomic profiles using GC×GC-TOFMS. The study was conducted as a part of an interlaboratory study led by the National Institute of Standards and Technology (NIST) in an effort to develop a human stool reference material for metabolomics and microbiome studies. All the samples used in the study were provided by the NIST. Only the data obtained from the GC×GC-TOFMS system by the authors are presented in this work with a particular focus on the data analysis, discussing the results comprehensively using various approaches.

## 2. Materials and Methods

### 2.1. Samples

Human whole stool was obtained from multiple volunteer donors by The BioCollective (TBC) (Denver, CO, USA). All whole-stool samples were collected after informed consent under approved Institutional Review Boards protocols at TBC. Stool samples were collected from 8 volunteer donors: 2 vegan females, 2 vegan males, 2 omnivore females, and 2 omnivore males. Attempts were made to match volunteers for each diet cohort by age, sex, and BMI so that diet would be the main difference between the two cohorts. The volunteers ranged between 20 and 65 for age and 18.5 and 29.9 for BMI and were all surveyed for health and diet. The donors were self-reported as healthy with no antibiotic use within 60 days of donation and no routine use of other medication. The samples were deposited into a BioCollectorTM and shipped overnight on an ice brick (the sample temperature was maintained at approximately 4 °C). Upon receipt, each sample was segmented into 30 g to 50 g portions, stored in specimen collection jars, and placed at −80 °C until processing. A portion of the first stool sample from each donor was subjected to pathogen screening for HIV, Hepatitis B, and Hepatitis C using the Biogates One Step Rapid Diagnostic Tests. The material was homogenized and aliquoted by TBC and preserved either in an aqueous form (100 mg wet stool/mL in water, stored frozen at −80 °C) or lyophilized (aliquoted at 100 mg wet stool/mL into lyophilization vials and freeze dried). All aliquots (lyophilized and aqueous) were shipped to the NIST on dry ice and placed immediately into −80 °C storage upon arrival (NIST report in preparation). The pooled human fecal samples of two diet cohorts (omnivores and vegans) stored in two preservation methods (lyophilization and frozen aqueous) were provided as Research-Grade Test Materials by the NIST. These four groups (2 diets × 2 storage conditions) were denoted as LV, LO, AV, and AO, representing lyophilized vegans, lyophilized omnivores, aqueous vegans, and aqueous omnivores, respectively, for the rest of the manuscript. For each of the four groups, three replicate sample vials were originally provided by the NIST. Each sample vial was analyzed in duplicate, rendering six replicates per group, resulting in a total of 24 analytical runs for the current study.

### 2.2. Chemicals

Methanol (>99.9%, HPLC grade, Millipore-Sigma, Oakville, ON, Canada) and ultrapure 18.2 MΩ deionized water, supplied from an Elga PURELAB Flex 3 system (VWR International, Edmonton, AB, Canada), were mixed to prepare an 80% methanol (*v*/*v*) extraction solvent. Toluene (>99.5%, ACS grade), pyridine (>99.9%, HPLC grade), anhydrous sodium sulfate (>99.0% ACS grade), and methoxyamine hydrochloride (98%) were obtained from Millipore-Sigma (Oakville, ON, Canada). *N*-Methyl-*N*-(trimethylsilyl) trifluoroacetamide + 1% chlorotrimethylsilane (MSTFA + 1% TMCS), purchased from Fisher Scientific (Ottawa, ON, Canada), was used as a derivatizing reagent. A total of 1 mg of ^13^C_4_ methylmalonic acid (Millipore-Sigma, Oakville, ON, Canada) was dissolved in 10 mL of deionized water to prepare an internal standard solution.

### 2.3. Sample Preparation

Upon receiving the samples from the NIST (frozen in dry ice), samples were stored at −80 °C until analysis. All samples in this study were thawed once on the day of analysis to ensure that they experienced the same number of freeze–thaw cycles (after initial preparation by the NIST). Aqueous samples were thawed at room temperature for one hour, followed by vortexing (Benchmark Scientific Benchmixer V2) for 3 min and centrifugation at 10,800× *g* for 10 min at 4 °C before aliquoting. A total of 12 mg of lyophilized samples and 400 µL of supernatants for aqueous samples were aliquotted into 2 mL centrifuge tubes (Fisher Scientific, Ottawa, ON, Canada). A 15 µL aliquot of the internal standard solution was spiked into each tube. Then, 450 µL and 600 µL of 80% methanol extraction solvent were added to the lyophilized samples and aqueous samples, respectively, followed by vortexing for 3 min and centrifugation at 10,800× *g* for 10 min at 4 °C. Next, 300 µL of the lyophilized and 600 µL of the aqueous supernatants were transferred into separate GC vials. The extracts were dried under a gentle stream of nitrogen at 50 °C. To the dried extracts, 100 µL of toluene dried with anhydrous sodium sulfate was added, and drying under nitrogen at 50 °C was repeated. Then, 50 µL of methoxyamine hydrochloride solution (20 mg/mL in pyridine) was added and incubated at 60 °C for 2 h. The samples were removed from heat and cooled for 5 min at room temperature. Next, 100 µL of MSTFA + 1% TMCS was added and incubated at 60 °C for 45 min. The samples were cooled for 5 min at an ambient temperature and then transferred into a GC vial with a fused glass insert (Chromatographic Specialties, Brockville, ON, Canada). All the samples were analyzed within 48 h from the completion of derivatization.

### 2.4. GC×GC-TOFMS Conditions

Two-dimensional chromatographic separations were performed on a LECO Pegasus 4D system (LECO, St. Joseph, MI, USA) equipped with a quad jet dual-stage modulator. A column set of 60 m × 0.25 mm; 0.25 µm df Rxi-5SilMS (Chromatographic Specialties, Brockville, ON, Canada) and 1.6 m × 0.25 mm; 0.25 µm df Rtx-200MS (Chromatographic Specialties, Brockville, ON, Canada) was used for the first and second dimension, respectively. A GERSTEL MPS autosampler (GERSTEL Inc., Linthicum, MD, USA), controlled using MAESTRO software, was used for the automated injection of 1 µL aliquots of sample in splitless mode. The main oven was programmed to start at 80 °C (4 min hold) followed by a ramp of 3.5 °C/min to 315 °C (10 min hold). The secondary oven and modulator temperature offset were constant at +10 °C relative to the main oven and +15 °C relative to the secondary oven, respectively. The modulation period was 2.50 s (0.60 s hot pulse and 0.65 s cold pulse time). Helium was used as the carrier gas at a corrected constant flow rate of 2 mL/min for the entire run. The transfer line and the ion source temperature were set at 250 °C and 200 °C, respectively. Mass spectra were collected over a mass range *m*/*z* 40–800 at an acquisition rate of 200 Hz. The electron impact energy of −70 eV and the detector voltage at an offset of −200 V relative to the tuning potential were used.

### 2.5. Data Processing and Analysis

All 24 individual GC×GC-TOFMS chromatograms were processed using ChromaTOF^®^ (v.4.72; LECO, St. Joseph, MI, USA) with the same initial data processing method described by Nam et al. [26] In brief, the initial method to process each chromatogram involves the exclusion of the column bleed region and setting the baseline offset to 0.9, and the expected peak widths to 12 s for the first dimension and 0.15 s for the second dimension, the peak-finding threshold of S/N to 100 searching against the peaks containing *m*/*z* 73 (trimethylsilyl fragment), with the minimum S/N ratio of 6 for the inclusion of sub-peaks. All detected peaks were searched against the NIST-MS 2017 Library mass spectral database.

The processed chromatograms were aligned using the Statistical Compare feature of ChromaTOF^®^. To obtain the basic statistics and the most predominant metabolites discussed in Section 3.1 and Section 3.2, each diet style and storage condition was aligned separately. For projection on PCA addressed in Section 3.3, all 24 samples from four groups were aligned together. To determine the distinguishing metabolites, combinations of two groups were aligned with the details explained in Section 3.3. The Fisher ratio was calculated from the Statistical Compare feature. For all alignments, the same parameters were used. Tolerances for retention time shift were set to ±6 modulation periods in the first dimension and 0.2 s for the second dimension, with a minimum similarity of 600 for a spectral match. The threshold of S/N 20 was set for peaks not found by the initial peak finding to be included. Only the peaks in a minimum of 5 samples or in a minimum of 50% of samples in a class were kept.

Each aligned peak table was then exported as a .csv file and imported into MATLAB^®^ 2020a. Principal Component Analysis (PCA) was performed using PLS_Toolbox 9.1 (Eigenvector Research Inc., Wenatchee, WA, USA). The Total Useful Peak Area (TUPA), representing the sum of the peak areas of the common peaks in all of the samples, was used as a normalization factor [35]. The compound class analysis and jitter plots described in Section 3.4 were generated using Microsoft Excel 2013.

## 3. Results and Discussion

### 3.1. General Comparison

**Figure 1:**
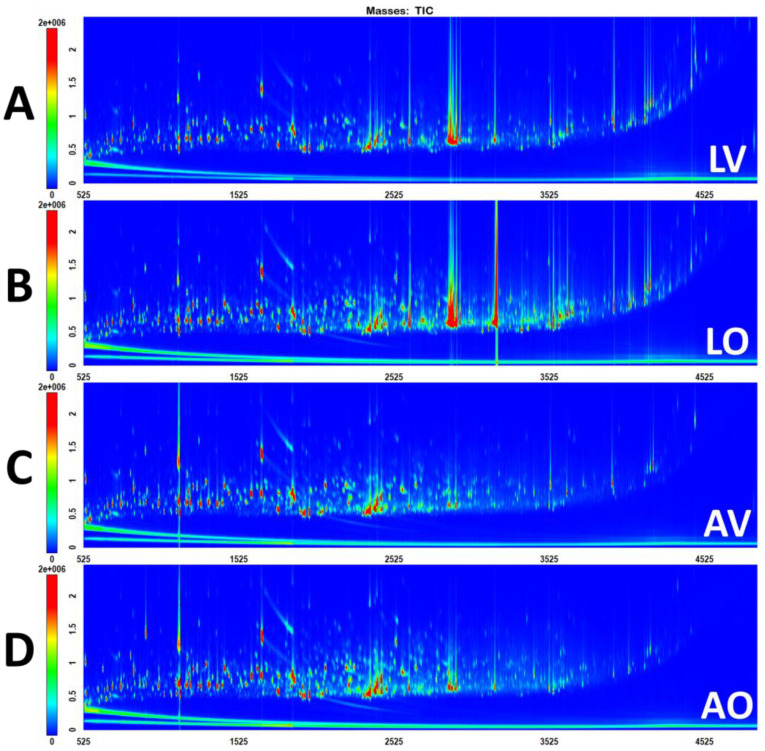
Representative GC×GC-TOFMS TIC of (**A**) lyophilized vegan, (**B**) lyophilized omnivore, (**C**) aqueous vegan, and (**D**) aqueous omnivore samples.

The representative GC×GC-TOFMS Total Ion Chromatograms (TICs) of human feces from two diets in two storage conditions are shown in Figure 1. The chromatograms of all six replicates for each condition were included in the Supplementary Information (SI) to demonstrate the reproducibility. It is noteworthy that the storage conditions had a more pronounced impact on metabolic profiles than the diets. From just visually screening the raw chromatograms, the differences in metabolic profiles for different storage conditions were perceivably apparent (Figure 1). Compared to the aqueous samples, the lyophilized samples had more intense peaks of saturated and unsaturated C18 fatty acids, which eluted in the region of 2850–2950 s in the first dimension as well as a busier later region of the chromatogram (above 3500 s), which is where bile acids and sterols elute. Comparing LV and LO, significant differences were not noted, other than LO displaying an intense peak in the region of 3180 s, which was unable to be identified due to the distortion of a peak shape owing to overloading. AV and AO indicated intensity differences for some peaks but did not exhibit apparent differences in terms of metabolic profiles using TIC.

For each sample, over 1000 peaks from a wide variety of compound classes were detected. Commonly observed compound classes included amino acids, fatty acids, carbohydrates, bile acids, sterols, tocopherols, and nucleosides. Quantitative results comparing four conditions are outlined in Table 1, summarizing the average values of six replicates for the total number of detected peaks, total peak area (TPA), and TUPA. For both lyophilized and aqueous samples, more peaks and higher signal intensities were observed in omnivores than vegans. The relative standard deviation (RSD) of the replicates was used to assess the reproducibility for each condition (Table 1), while the TPA and TUPA for each replicate are included in the Supplementary Materials (Table S1). The highest RSDs for both TPA and TUPA were observed with the lyophilized vegan samples, while lyophilized omnivore samples had the second highest RSDs for both TPA and TUPA. For aqueous samples, less than 4% of RSDs were obtained for both vegans and omnivores. This indicates that the extraction from aqueous feces appears to be more reproducible than lyophilized fecal samples; this is a result consistent with the findings reported by Cui et al., who also noted the direct extraction of the fecal slurry was more reproducible than lyophilized samples using proton nuclear magnetic resonance (^1^H NMR) spectroscopy [33].

### 3.2. Most Abundant Metabolites

To determine the most abundant metabolites for each condition, the relative area of analytes was calculated by dividing the area of each peak by the total peak area of the condition. The relative area of each analyte was then averaged for six replicates. The metabolites with a relative area above 1% that were consistently detected in all six replicate samples are listed in descending order in Table 2. The analytes were tentatively identified based on the retention indices and mass spectral matches against the library (SI). A compound name was not assigned for the analytes with a mass spectral match score of less than 600. Detailed information on the most abundant metabolites for each group is included in the SI. For vegans, 18 compounds for lyophilized and 16 compounds for aqueous samples had a relative abundance of greater than 1%, making up 33.90% and 34.99% of the total peak area, respectively. For omnivores, 13 compounds for lyophilized and 18 compounds for aqueous samples had a relative abundance above 1%, which comprised 29.09% and 34.36% of the total peak area, respectively (Table 2).

L-5-Oxoproline was detected as the most abundant compound in all four groups, with a similar relative abundance across the conditions ranging from 5.55% to 5.96%. Pyroglutamic acid and tyrosine were also at a high level in all four conditions, with the relative abundance ranging from 2.32% to 3.46% and 1.18% to 1.82%, respectively. 7H-purine was a notable compound, which existed in high concentrations in the three groups except in the lyophilized vegan samples, where the relative abundance was only 0.02%. Other predominant metabolites commonly present at high levels in both types of vegan samples included leucine and glycine. Phenylalanine, proline, and ribose were found to be the abundant metabolites with a relative abundance above 1% in both lyophilized and aqueous omnivore samples. On the other hand, sugar compounds, including ribose and arabinose, were detected at high levels in the lyophilized samples of the two diets. Alanine, glycine, valine, ribose, and xylose were present in high levels for the aqueous samples of both diets.

### 3.3. Chemometrics Analysis and Feature Selection

**Figure 2:**
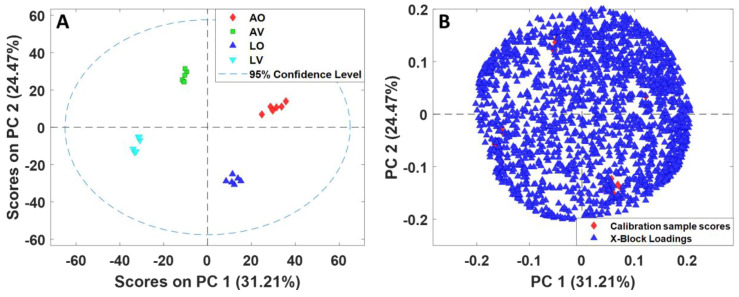
(**A**) PCA scores plot of all 24 samples with 1884 variables normalized by TUPA (**B**) biplot.

The alignment of all 24 samples (four groups of six replicates) resulted in 1884 variables. PCA was employed for the dataset of 24 samples with 1884 variables to assess which samples were similar and different from each other. The PCA scores plot using the first two principal components (PCs) and the biplot are shown in Figure 2. All four groups exhibited distinct clustering well away from one another, signifying that they were unquestionably different. In total, 31.21% and 24.47% of the variations in the dataset were captured by the first (PC1) and second (PC2) principal components, respectively (Figure 2A). The separation between omnivores and vegans was explained by PC1, whereas the differences between lyophilized and aqueous samples were described by PC2.

**Figure 3:**
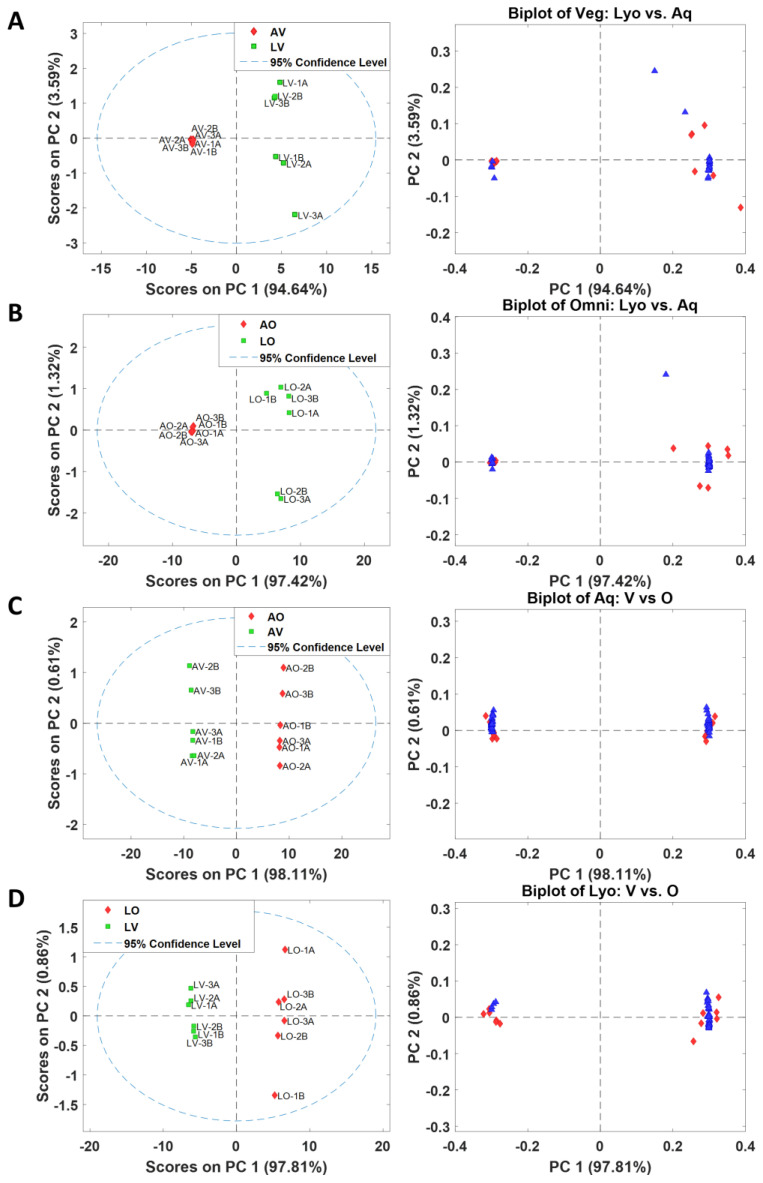
PCA scores plot and biplot using the selected variables for each dataset (**A**) LV vs. AV (28 variables), (**B**) LO vs. AO (54 variables), (**C**) AV vs. AO (79 variables), and (**D**) LV vs. LO (41 variables). In the biplots, red diamonds represent samples and blue triangles represent variables.

Although the PCA scores plot of 24 appears to be uncomplicated, with significant separations between classes using all 1884 variables without feature selection, it is challenging to discern which features are responsible for separating groups, as depicted in the biplot shown in Figure 2B. To identify relevant features distinguishing between groups, feature selection was performed based on the Fisher ratio. Each of the four groups is unique, as represented in the raw chromatograms (Figure 1) and the PCA scores plot (Figure 2A); the different groups could not be combined to extract distinguishing features. For example, it is not appropriate to put lyophilized and aqueous omnivores together into one class versus lyophilized and aqueous vegans as the other class to find the metabolites that distinguish between omnivores and vegans. LV and AV are considerably different and would result in a vast within-class variation if they were combined as one group, which would cause an erroneous result for feature selection. Therefore, four separate feature selection processes were required as follows: (1) LV vs. AV, (2) LO vs. AO, (3) AV vs. AO, and (4) LV vs. LO. The chromatograms for each feature selection grouping were re-aligned with the inclusion of 12 samples (6 replicates each for 2 classes) only, which resulted in 1706, 1790, 1668, and 1587 peaks, respectively. The Fisher ratios were calculated for each set, and peaks were sorted in descending order according to the Fisher ratio. The analytes with a Fisher ratio above 300 and present in more than 2/3 of the samples were selected. As a result, 28, 54, 79, and 41 peaks were selected. The selected features were tentatively identified based on the retention indices and the mass spectral matches. Detailed information on the selected features for all four combinations is included in the SI. The PCA analysis was newly performed using only the selected features, and the scores plot and biplot are shown in Figure 3. A clear separation between the two groups was attained with PC1 for all four cases, capturing above 94% of total variations.

### 3.4. Analysis via Compound Classes

The complexity of feces with contrasting chemistries of a wide variety of compound classes creates the need to perform analyses via compound classes to evaluate how different families of compounds respond under the different sample storage conditions [26]. Thus, a group-type study was conducted by organizing the metabolites into compound classes and summing the signal intensities of the same class members. The total intensity for each class was then normalized to the average of all 24 samples. The compound classes for the group-type analysis in this work included fatty acids, amino acids, tocopherols, sterols, bile acids, and nucleosides. Detailed information on the individual members belonging to the compound classes is included in the SI.

**Figure 4:**
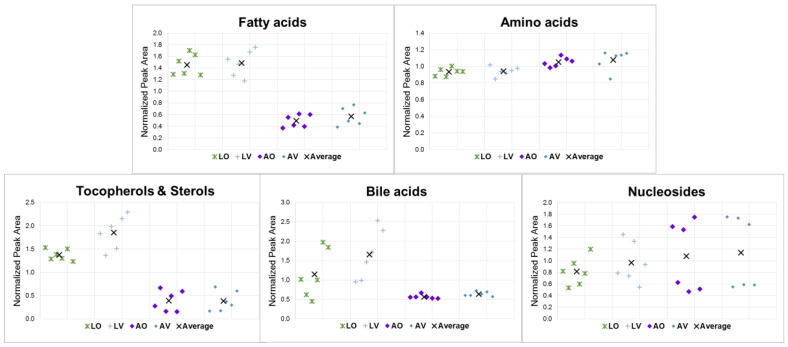
Analysis via compound classes depicted using jitter plots (*n* = 6).

Figure 4 shows the jitter plots of five classes of compounds comparing the four conditions. The same plots with the sample labels to indicate different replicates that are included in the Supplementary Materials (Figure S5). In general, more considerable within-class variations were found with lyophilized samples than with aqueous samples, except for nucleosides. This is consistent with the results obtained for the overall comparison discussed in Section 3.1. The additional steps involved in the lyophilization process while risking the loss of some volatiles may have contributed to the added variations. Larger variations were observed for the class of nucleosides compared to the other classes due to the low intensities and fewer number of the members of the compounds that belong to the class. For fatty acids, bile acids, tocopherols, and sterols, the lyophilized samples had higher intensities than the aqueous samples. This result is thought to be attributed to the wet and slushy nature of aqueous samples, which makes it difficult to extract fatty and bulky compounds. In contrast, lyophilization makes samples more prone to release metabolites, resulting in the intensification of signals [26]. Overall, the group-type analysis also revealed similar patterns for the samples in the same storage conditions, which may be interpreted as the storage conditions having a more significant impact on the metabolic profiles than diet styles.

## 4. Conclusions

In the current work, the metabolic profiles of human feces from two different diet styles stored in two conditions were analyzed using a high-separation-power analytical instrument, GC×GC-TOFMS. The acquired data were analyzed using various approaches, suggesting ideas that can lead to a meaningful interpretation of the large number of GC×GC-TOFMS metabolomics data that are not straightforward to handle. The results from the current study altogether indicate that the sample storage condition is a remarkable factor that has a massive impact on the metabolic profiles of feces, which even outperforms another well-known significant factor, diet style. These findings would benefit microbiome and metabolomics communities for the development and implementation of a fecal reference material along with the standardization of preanalytical aspects. It would also contribute towards the more widespread use of GC×GC-TOFMS within the field.

## Figures and Tables

**Table 1:** Comparison among feces from different diets and sample storage conditions (*n* = 6).

		Vegans			Omnivores		
		Number of Peaks	Average	RSD (%)	Number of Peaks	Average	RSD (%)
Lyophilized	TPA	1417	1.93 × 10^9^	15.2	1544	2.44 × 10^9^	5.52
	TUPA	1189	1.78 × 10^9^	15.8	1279	2.28 × 10^9^	6.27
Aqueous	TPA	1506	2.46 × 10^9^	1.64	1595	2.73 × 10^9^	3.38
	TUPA	1236	2.14 × 10^9^	1.94	1306	2.40 × 10^9^	3.97

**Table 2:** Most abundant metabolites with an area percent greater than 1% (*n* = 6).

	Vegans		Omnivores	
	Compound	Rel. ab (%)	Compound	Rel. ab (%)
**Lyophlized**	L-5-Oxoproline	5.60	L-5-Oxoproline	5.96
	d-Ribose	3.46	7H-purine	3.86
	Phenylalanine	2.43	Pyroglutamic acid	3.46
	Pyroglutamic acid	2.32	d-Ribose	2.68
	L-Threonine	2.24	D-Arabinose	2.23
	L-Aspartic acid	1.93	Phenylalanine	1.87
	Analyte 52	1.86	L-Proline	1.54
	L-Leucine	1.66	L-Tyrosine	1.44
	Propylene glycol	1.54	2-Monolinolenin	1.39
	D-(−)-Rhamnose	1.40	N-Methyl-à-aminoisobutyric acid	1.30
	9,12-Octadecadienoic acid	1.40	5-Hydroxyindoleacetic acid	1.28
	DL-Arabinose	1.40	L-Valine	1.07
	L-Tyrosine	1.18	Tricarballylic acid	1.02
	Glycine	1.17		
	Butanedioic acid	1.10		
	D-(+)-Xylose	1.09		
	Uric acid	1.05		
	d-Glucose	1.07		
	**Total**	**33.90**	**Total**	**29.09**
**Aqueous**	L-5-Oxoproline	5.79	L-5-Oxoproline	5.55
	7H-purine	3.91	Pyroglutamic acid	3.28
	Pyroglutamic acid	3.31	7H-purine	3.05
	Serine	2.51	Phenylalanine	2.52
	L-Alanine	2.49	Malic acid	2.31
	d-Ribose	2.21	L-Alanine	2.06
	Glycine	1.84	L-Tyrosine	1.81
	L-Tyrosine	1.82	d-Ribose	1.64
	L-Leucine	1.67	Glycine	1.55
	D-(−)-Rhamnose	1.54	L-Proline	1.38
	Uric acid	1.50	D-(+)-Cellobiose	1.36
	Analyte 48	1.35	L-Isoleucine	1.25
	Propylene glycol	1.34	N-Methyl-à-aminoisobutyric acid	1.22
	L-(−)-Fucose	1.31	Analyte 50	1.19
	L-Valine	1.30	L-Valine	1.17
	D-(+)-Xylose	1.11	Galactaric acid	1.01
			Monomethylphosphate	1.00
			D-(+)-Xylose	1.00
	**Total**	**34.99**	**Total**	**34.36**

## Data Availability

The data presented in the study are available in the Supplementary Materials.

## Supplementary Materials

The following supporting information can be downloaded at: https://www.mdpi.com/article/10.3390/metabo13070828/s1, Figure S1: TIC of six replicates for the lyophilized vegan samples; Figure S2: TIC of six replicates for the lyophilized omnivore samples; Figure S3: TIC of six replicates for the aqueous vegan samples; Figure S4: TIC of six replicates for the aqueous omnivore samples; Figure S5: Analysis by compound classes depicted using jitter plots with sample labels; Table S1: Comparison among feces from different diets and sample storage conditions; Table S2: Most abundant metabolites; Table S3: Distinguishing metabolites; Table S4: Group type analysis.
