# Supplementary material for: Untargeted Metabolomic Profiling of Aqueous and Lyophilized Pooled Human Feces from Two Diet Cohorts Using Two-Dimensional Gas Chromatography Coupled with Time-of-Flight Mass Spectrometry
Source: Metabolites. 2023 Jul 7;13(7):828. doi: 10.3390/metabo13070828 (PMC10383202; doi:10.3390/metabo13070828)
Supplement: Supplementary file 1 [file metabolites-13-00828-s001.zip › NIST SI.pdf]

Untargeted metabolomic profiling of aqueous and lyophilized pooled human feces from two diets cohorts using GC×GC-TOFMS

Seo Lin Nam [1], Kieran Tarazona Carrillo [1], A. Paulina de la Mata [1], James Harynuk [1*]

[1] Department of Chemistry, University of Alberta, Edmonton, Alberta, Canada;

seolin@ualberta.ca (S.L.N.); ktarazon@ualberta.ca (K.T.C.); delamata@ualberta.ca (A.P.d.l.M.)

[*] Author to whom correspondence should be addressed; E-Mail: james.harynuk@ualberta.ca;

Tel.: +1-780-492-8303 (ext. 123); Fax: +1-780-492-8231. Department of Chemistry University of Alberta, Edmonton, Alberta, T6G 2G2, Canada

Supporting Information 1 –Total Ion Chromatograms (TIC) of six replicates (three sample replicates analyzed in duplicates) per condition demonstrating the reproducibility of the GC×GC-TOFMS fecal metabolomics workflow

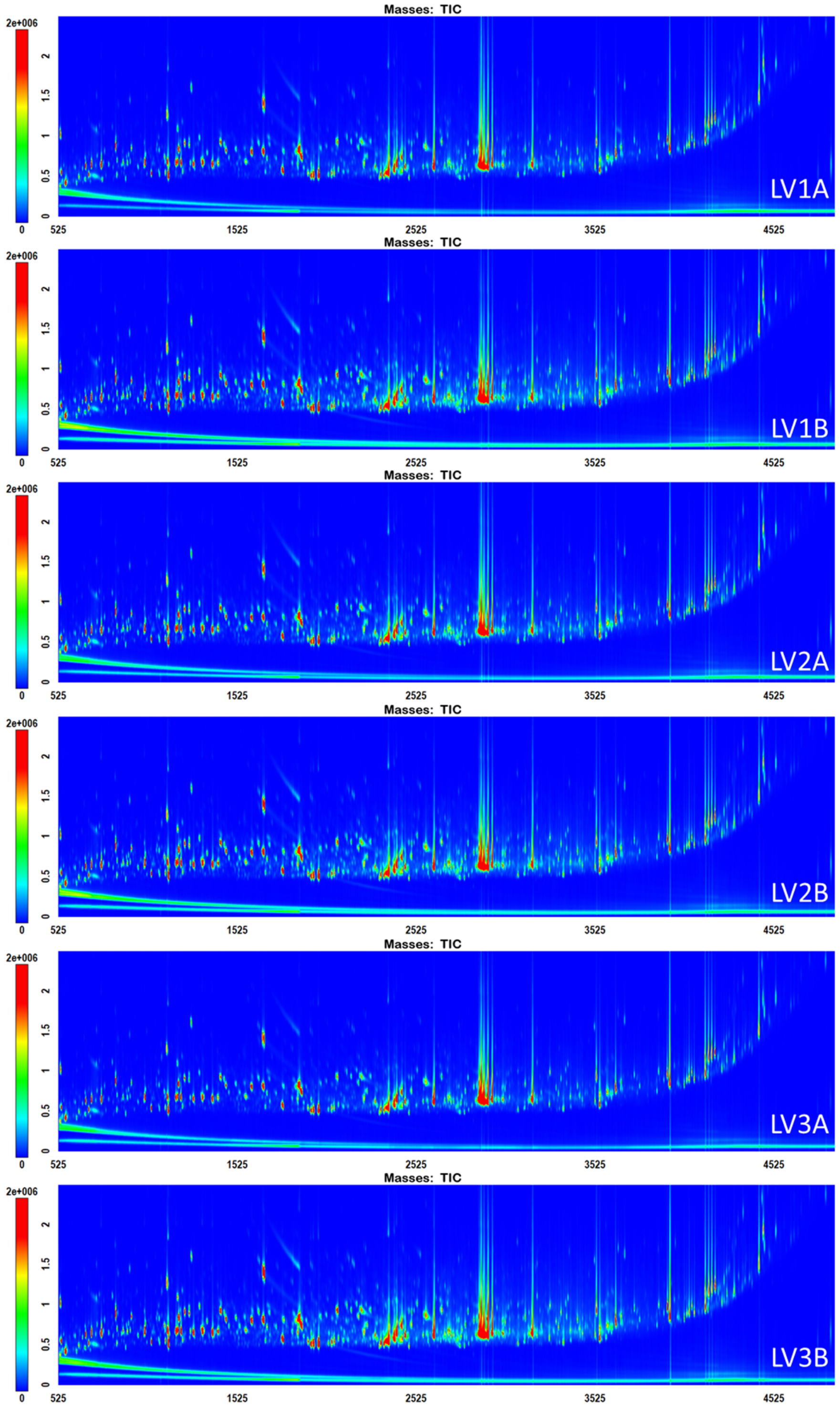

Figure S1 TIC of six replicates for the lyophilized vegan samples

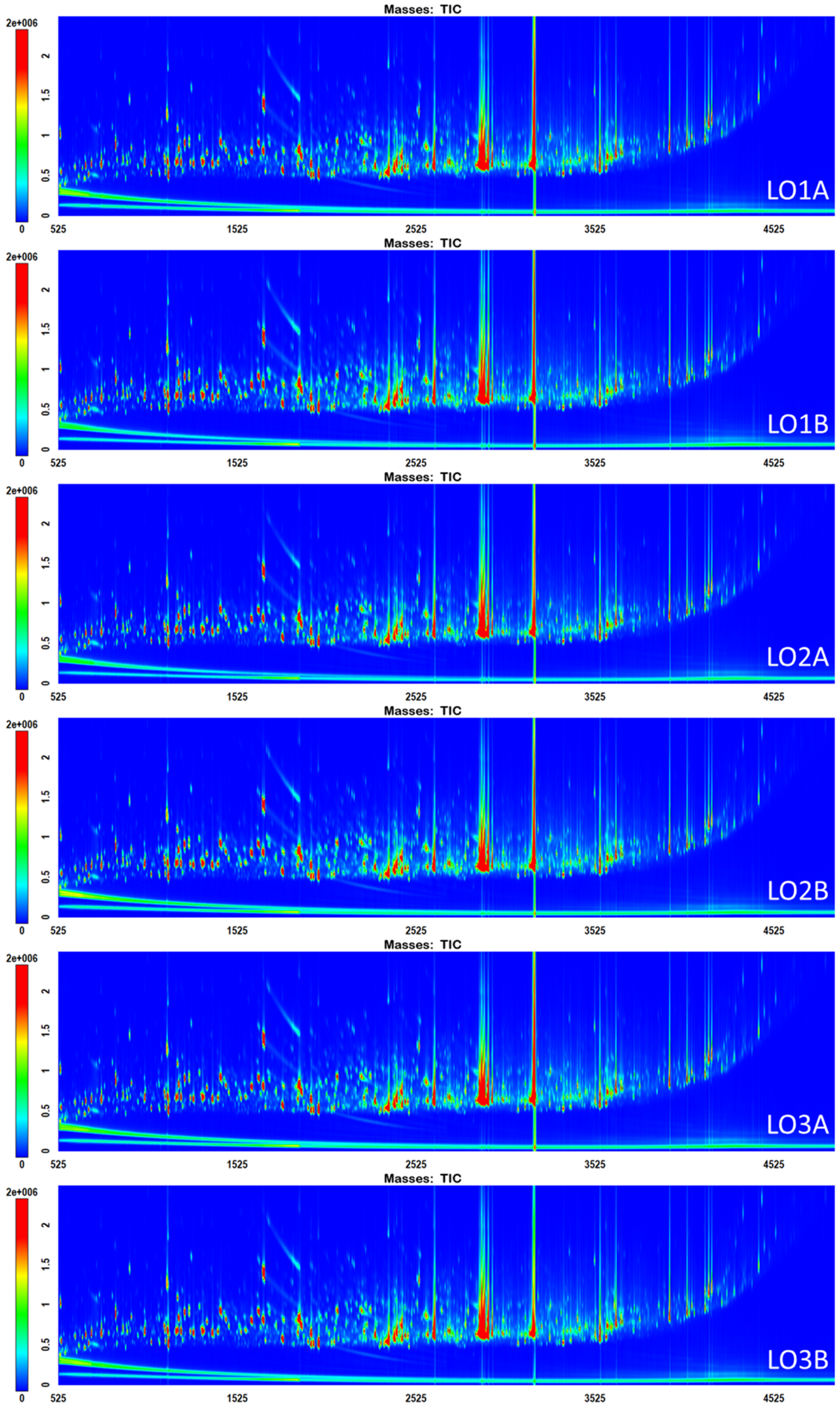

Figure S2 TIC of six replicates for the lyophilized omnivore samples

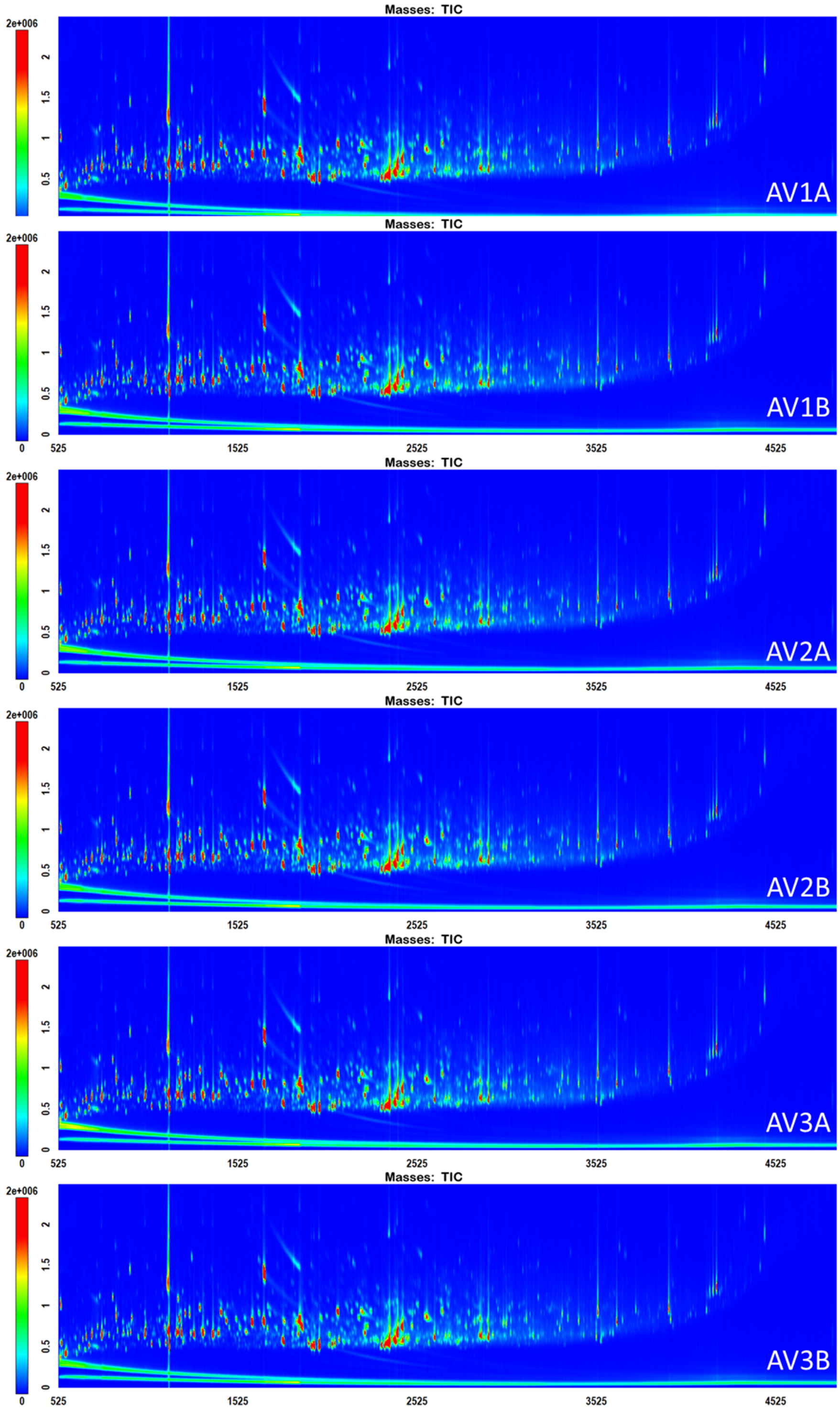

Figure S3 TIC of six replicates for the aqueous vegan samples

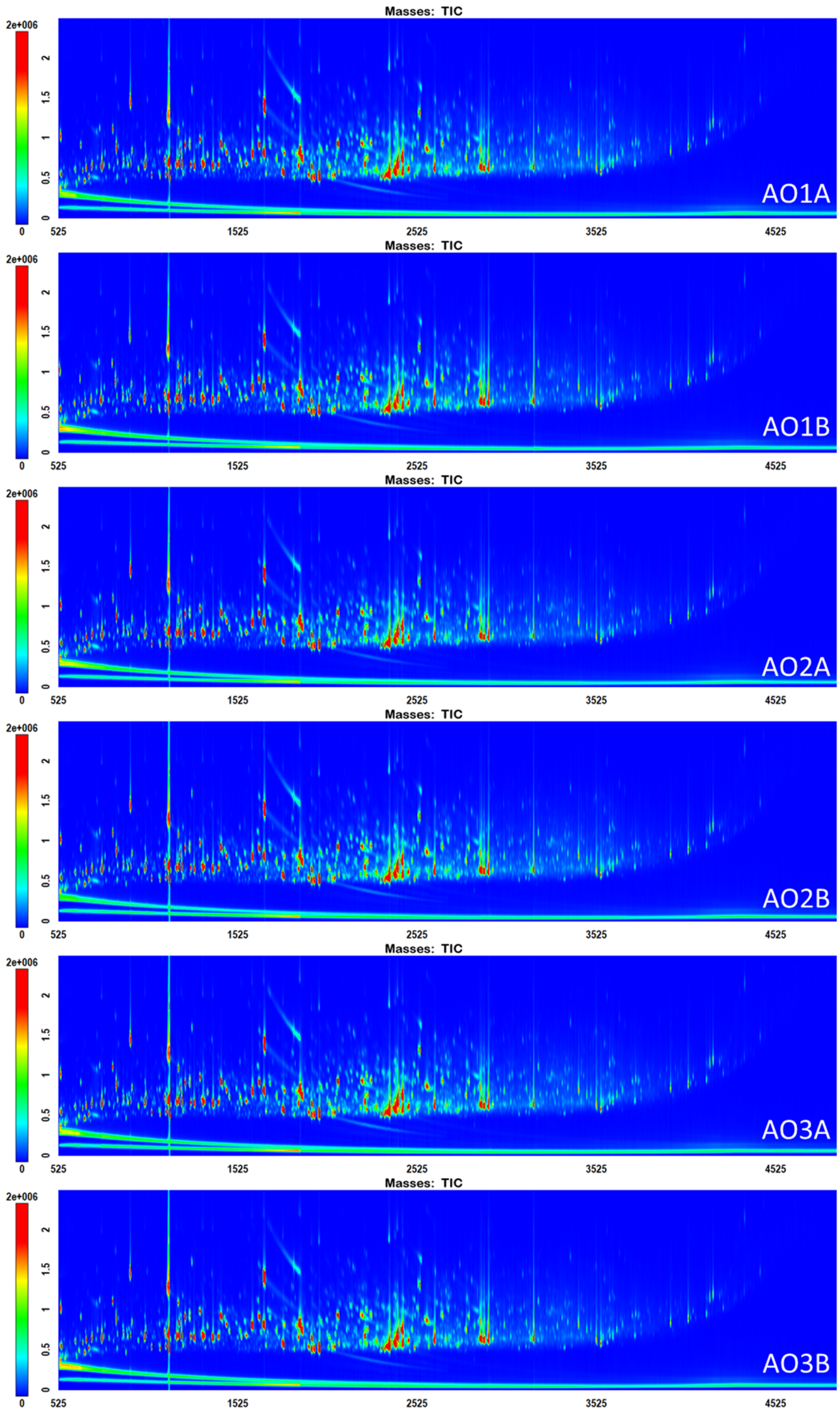

Figure S4 TIC of six replicates for the aqueous omnivore samples

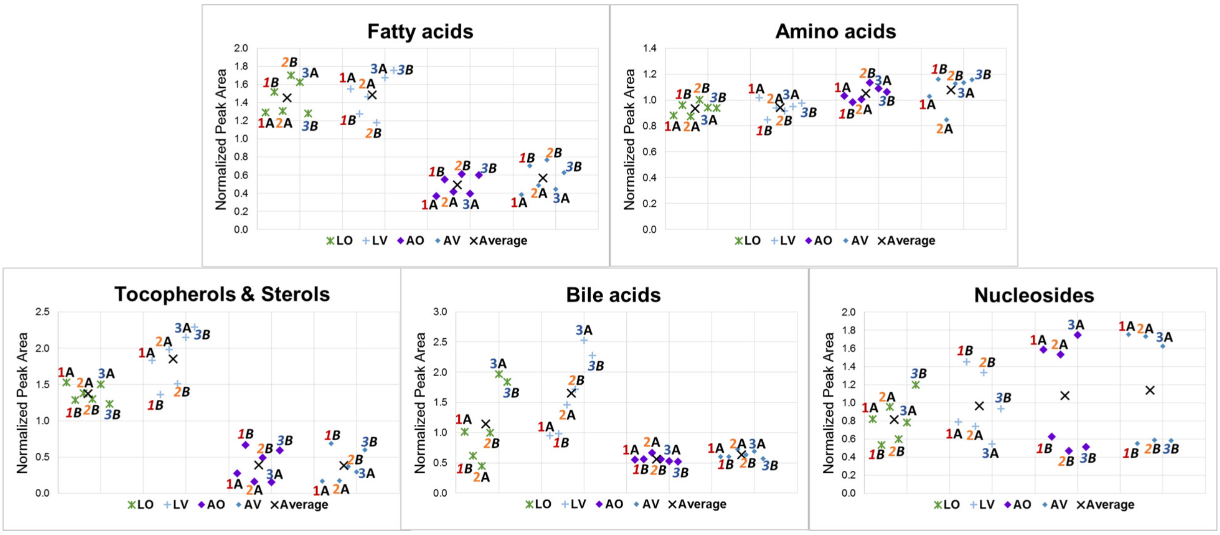

Figure S5 Analysis by compound classes depicted using jitter plots with sample labels. The sample labels were color-coded by red: tube 1, yellow: tube 2, blue: tube 3. A indicates the first duplicate whereas B represents the second duplicate and was italicized.

Table S1. Comparison among feces from different diets and sample storage conditions *(n = 6 per group)*.

		Vegans			Omnivores		
**Lyophilized**		**LV-1**	**LV-2**	**LV-3**	**LO-1**	**LO-2**	**LO-3**
**TPA**	**A**	1.63×10^9	1.88×10^9	1.54×10^9	2.33×10^9	2.27×10^9	2.47×10^9
	**B**	2.16×10^9	2.13×10^9	2.24×10^9	2.66×10^9	2.48×10^9	2.43×10^9
	**Average**	**1.90×10^9**	**2.00×10^9**	**1.89×10^9**	**2.50×10^9**	**2.37×10^9**	**2.45×10^9**
**TUPA**	**A**	1.54×10^9	1.72×10^9	1.38×10^9	2.20×10^9	2.08×10^9	2.32×10^9
	**B**	1.99×10^9	1.96×10^9	2.10×10^9	2.50×10^9	2.34×10^9	2.25×10^9
	**Average**	**1.76×10^9**	**1.84×10^9**	**1.74×10^9**	**2.35×10^9**	**2.21×10^9**	**2.29×10^9**
**Aqueous**		**AV-1**	**AV-2**	**AV-3**	**AO-1**	**AO-2**	**AO-3**
**TPA**	**A**	2.54×10^9	2.46×10^9	2.43×10^9	2.76×10^9	2.71×10^9	2.86×10^9
	**B**	2.44×10^9	2.45×10^9	2.45×10^9	2.66×10^9	2.78×10^9	2.59×10^9
	**Average**	**2.49×10^9**	**2.46×10^9**	**2.44×10^9**	**2.71×10^9**	**2.74×10^9**	**2.73×10^9**
**TUPA**	**A**	2.17×10^9	2.16×10^9	2.18×10^9	2.51×10^9	2.31×10^9	2.48×10^9
	**B**	2.11×10^9	2.15×10^9	2.07×10^9	2.37×10^9	2.45×10^9	2.27×10^9
	**Average**	**2.14×10^9**	**2.15×10^9**	**2.13×10^9**	**2.44×10^9**	**2.38×10^9**	**2.38×10^9**

Each group (LV, LO, AV, AO) contains three replicate sample vials provided by the NIST, indicated as 1, 2 and 3. Each replicate sample vial was analyzed in duplicate, which was indicated as A and B. For example, the second duplicate of the replicate vial 3 of the lyophilized vegan was denoted as LV-3B.
